# Vagus Nerve Stimulation as a Potential Therapy in Early Alzheimer’s Disease: A Review

**DOI:** 10.3389/fnhum.2022.866434

**Published:** 2022-04-29

**Authors:** Mariana Vargas-Caballero, Hannah Warming, Robert Walker, Clive Holmes, Garth Cruickshank, Bipin Patel

**Affiliations:** ^1^School of Biological Sciences, University of Southampton, Southampton, United Kingdom; ^2^Memory Assessment and Research Centre, Southern Health Foundation Trust, Southampton, United Kingdom; ^3^Queen Elizabeth Hospital Birmingham, University of Birmingham, Birmingham, United Kingdom; ^4^ElectronRx, Cambridge, United Kingdom

**Keywords:** vagus, MCI, Alzheimer, memory, plasticity, norepinepherine, noradrenaline, vagal

## Abstract

Cognitive dysfunction in Alzheimer’s disease (AD) is caused by disturbances in neuronal circuits of the brain underpinned by synapse loss, neuronal dysfunction and neuronal death. Amyloid beta and tau protein cause these pathological changes and enhance neuroinflammation, which in turn modifies disease progression and severity. Vagal nerve stimulation (VNS), *via* activation of the locus coeruleus (LC), results in the release of catecholamines in the hippocampus and neocortex, which can enhance synaptic plasticity and reduce inflammatory signalling. Vagal nerve stimulation has shown promise to enhance cognitive ability in animal models. Research in rodents has shown that VNS can have positive effects on basal synaptic function and synaptic plasticity, tune inflammatory signalling, and limit the accumulation of amyloid plaques. Research in humans with invasive and non-invasive VNS devices has shown promise for the modulation of cognition. However, the direct stimulation of the vagus nerve afforded with the invasive procedure carries surgical risks. In contrast, non-invasive VNS has the potential to be a broadly available therapy to manage cognitive symptoms in early AD, however, the magnitude and specificity of its effects remains to be elucidated, and the non-inferiority of the effects of non-invasive VNS as compared with invasive VNS still needs to be established. Ongoing clinical trials with healthy individuals and patients with early AD will provide valuable information to clarify the potential benefits of non-invasive VNS in cognition and AD. Whether invasive or non-invasive VNS can produce a significant improvement on memory function and whether its effects can modify the progression of AD will require further investigation.

## Introduction

Alzheimer’s disease (AD) is the most common form of dementia, with prevalence expected to increase globally from 50 million in 2019 to 152 million in 2050 (Patterson, 2018). The defining features of AD are neurofibrillary tangles and senile plaques composed of tau protein and amyloid beta, respectively (Nelson et al., 2012). Pathological staging of tau tangle formation suggests that AD starts in the trans-entorhinal region and progresses from the entorhinal and hippocampal regions to full blown neocortical pathology (Braak and Braak, 1995). Magnetic resonance imaging (MRI) analyses show an early reduction in hippocampal volume correlating with memory deficits in AD patients (Fox et al., 1998). Cognitive decline accelerates once the pathology invades the neocortex (Nelson et al., 2012), and prominent atrophy of the temporal lobe is evident (Theofilas et al., 2015).

In moderate AD, more than 50% of synapses have been lost in hippocampal subfields including CA1 and dentate gyrus (Scheff and Price, 2003; Scheff et al., 2006). Disruption of the brain microenvironment and neuronal circuits is caused by complex effects of amyloid (Aβ) and tau on glial and neuronal populations (Strooper and Karran, 2016). Mechanistic and functional studies in rodents have helped to model the earlier phases of the disease (Strooper and Karran, 2016). These have shown that Aβ and aberrant tau protein disrupt neural activity (Tamagnini et al., 2015), impair synaptic plasticity (Walsh et al., 2002; Shipton et al., 2011; Fá et al., 2016; Sri et al., 2019), cause the loss of synaptic contacts (Shankar et al., 2007; Shipton et al., 2011; Klyubin et al., 2012; Spires-Jones and Hyman, 2014; Cummings et al., 2015; Sri et al., 2019) and impair memory (Cleary et al., 2005; Shankar et al., 2008). Analyses in AD mouse models and human cells suggest that microglia are chronically activated or “primed” by the presence of tau and Aβ aggregates (Paresce et al., 1996; Perry and Holmes, 2014). This chronic activation results in an over-secretion of proinflammatory cytokines including Interleukin-1 (IL-1), Interleukin-6 (IL-6) and tumour necrosis factor alpha (TNF-α) which contributes to synapse and neuron loss, accelerating disease progression. Strategies that protect synapses or reduce pro-inflammatory cytokine production may slow disease progression or preserve memory functions in AD (Selkoe, 2002; Heneka et al., 2015; Strooper and Karran, 2016). Although several therapies have been approved to treat AD and numerous trials are still underway to target Aβ, tau, inflammation, synaptic and neuronal loss in AD, no therapy yet has shown to robustly delay the onset of the disease or alter its progression.

Neuromodulation *via* targeted stimulation of neural pathways has been explored as an attractive route to regulate cognition (Vonck et al., 2014), including brain stimulation for AD which encompasses non-invasive techniques through to deep brain stimulation (DBS) (Hansen, 2014). Deep brain stimulation has shown some successes in reducing the rate of hippocampal atrophy and increasing brain connectivity and memory, but is highly invasive and as such carries risks of major surgery including bleeding, infection, haematoma and epilepsy, reviewed in Luo et al. (2021). The vagal nerve provides extensive sensing and signalling to and from visceral organs as a major component of the parasympathetic nervous system and provides an essential modulatory role within the CNS. Here, we review the evidence that vagal nerve stimulation (VNS) modulates both neuronal and neuroglial function that has the potential to provide neuroprotection in AD. Direct invasive VNS can provide a robust response, however, this is contrasted with complications arising from the surgical procedure (such as infection and vocal cord palsy, *v.i.*) although these are, in contrast, much smaller than complications associated with DBS procedures. Non-invasive transcutaneous vagal nerve stimulation (tVNS) is also effective in eliciting brain signalling, and ongoing clinical trials using tVNS will shed light on the effectiveness of this therapy and whether it can have a positive impact on cognitive function or ultimately modify the trajectory of AD.

## Locus Coeruleus Modulation of Neural and Glial Cells Following Vagus Stimulation

**FIGURE 1:**
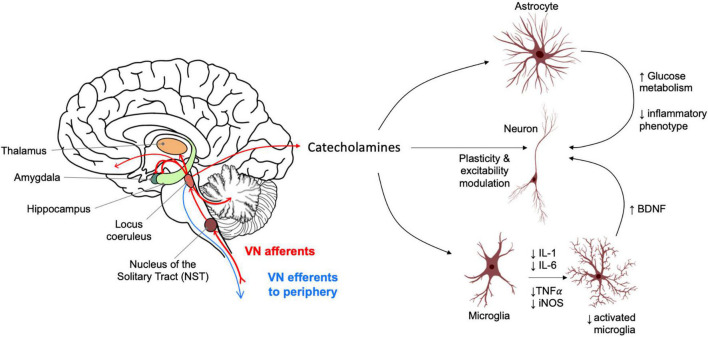
Vagus nerve afferent activity causes catecholamine release from the locus coeruleus into widespread brain regions. These include areas relevant for memory and highly impacted in Alzheimer’s disease such as the hippocampus. Norepinephrine (NE) acts on astrocytes and neuroglia, influencing an anti-inflammatory profile and neurotrophic support for neurons. NE also acts directly on neuronal populations to modulate synaptic plasticity and function with distinct effects depending on brain regions and neuron sub-types. LC activation also causes release of dopamine in the hippocampus which modulates neuronal plasticity and excitability and has a role in the consolidation of “everyday” type memory.

Vagus nerve efferents target a wide range of organs including the heart, lungs, gastrointestinal system, many glands and smooth muscle. However, the majority (∼80%) of vagal nerve fibres are afferent. A large proportion of these sensory fibres converge onto the spinal trigeminal nuclei of the medulla and the nucleus of the solitary tract (NTS). A monosynaptic projection from the NTS regulates the activity of the locus coeruleus (LC) which provides the sole source of norepinephrine (NE) within the brain (Janitzky, 2020) (Figure 1).

Vagal nerve stimulation (VNS) leads to a stimulation-intensity dependent increase in extracellular concentrations of NE in the hippocampus and cerebral cortex of rats, released from the LC (Roosevelt et al., 2006; Shen et al., 2012). Unmyelinated projections of the LC can communicate monosynaptically or by volume transmission, whereby neurotransmitters are released from varicosities along the axon that do not make contact with other synapses. At these varicosities NE diffuses into the surrounding space where it may act on neurons and glial cells (Feinstein et al., 2016). LC firing generates tonic or phasic release of NE; tonic firing is related to states of sleep and wakefulness, with a frequency of 1–3 Hz whilst awake (Howells et al., 2012), and helps to gate environmental inputs, while phasic firing occurs on encountering sensory inputs and during tasks that require attention, such as investigating a novel object.

In advanced AD, there is an evident degeneration of LC neurons (Bondareff et al., 1981). It has been proposed that early damage to the LC in the preclinical phase of AD may result in abnormally high tonic activity of the LC (Elman et al., 2017) which can impair phasic LC discharge, and that vagal stimulation may provide an avenue to restore phasic LC firing as suggested from research in rats (Janitzky, 2020).

## Vagus Nerve Stimulation

Vagal nerve stimulation (VNS) is currently used to treat refractory epilepsy that does not respond to pharmaceutical interventions in patients unsuitable for resective surgery, and for treatment-resistant depression. The first surgically implanted (i) VNS device was approved by the FDA (1997) to reduce the frequency, severity and length of seizures. iVNS devices are implanted in the chest under the clavicle, and cuff wires wrapped around the cervical vagus nerve trunk provide direct stimulation. Technology for other neuromodulation devices has moved faster than VNS; clinically approved devices for epilepsy include the brain-responsive neurostimulation (RNS) system which can deliver fine-tuned electrical stimulation in response to specific epileptiform activity (Jarosiewicz and Morrell, 2021), and two closed-loop devices have been FDA-approved for deep brain stimulation, and one for spinal cord stimulation (Fleming et al., 2020).

Although well tolerated, invasive VNS implantation and management can have side effects in approximately 10–30% of patients (Morris and Mueller, 1999). Surgical complications have been reported to occur in 9–17% of patients (Kahlow and Olivecrona, 2013; Révész et al., 2016) and these include hematoma, superficial or deep infection, and vocal cord palsy. Post-implantation issues after one year include hoarseness, paraesthesias, headache and shortness of breath. In addition to biological effects there is the risk of technical issues reported in 4–17% of patients (Kahlow and Olivecrona, 2013; Révész et al., 2016) such as lead fracture, disconnection, spontaneous turn-off, stimulator malfunction, battery or electrode failure and lead breakage, which require repeat surgery to correct. These complications have hampered the development of VNS use into patient groups outside epilepsy and depression as they imply further cost and readmissions.

To circumvent complication rate with iVNS, non-invasive devices have been developed and are currently being tested in clinical trials. However, clinical information on iVNS performance in the context of randomised control trials would be helpful to directly compare it with less or more invasive procedures (transcutaneous VNS or DBS, respectively) and weigh the risks against the benefits it could afford.

Transcutaneous VNS (tVNS) can be applied through locations on the ear (auricular) or in the neck (cervical) (Yap et al., 2020). There is functional MRI (fMRI) evidence that both tVNS and iVNS activate the same afferent vagal projection sites (Butt et al., 2020). Both techniques modulate brain activity by activating both afferent and efferent vagus nerve fibres (Clancy et al., 2013; Colzato and Beste, 2020) and have shown to cause an increase in salivary alpha amylase concentrations (Warren et al., 2019). However, mixed effects on the psychophysiological effects of vagus nerve responses have been reported. Whilst pupil size and P3 amplitude -an event-related potential elicited during decision making- are modulated by iVNS and in physiological VN responses (Burger et al., 2020), they are influenced by tVNS in some studies (Ventura-Bort et al., 2018; Sharon et al., 2021), but not in others (Warren et al., 2019). These observations suggest that although there is potential for tVNS to mimic iVNS, it is possible that the tVNS effect may be lower given the lack of direct stimulation of the vagus nerve, which is deep-seated in the neck within the carotid sheath.

## Vagus Nerve Stimulation: Anti-Inflammatory and Systemic Effects

Microglia and astrocytes contribute to normal brain function. Microglia have a high expression of α2 and β1 adrenoreceptors, and NE promotes BDNF production in these cells. This signalling has been shown to be essential in learning-related synapse formation in mice (Parkhurst et al., 2013). LC varicosities are highly associated with perivascular astrocyte end feet, exposing astrocytes to NE through volume transmission. Astrocytes support metabolic function of neurons and uptake of glutamate, which are enhanced by the activation of α1, α2 and β1 adrenergic receptors (O’Donnell et al., 2012). In the dentate gyrus of the hippocampus in mice, glycogen phosphorylase activation is enhanced by NE, supporting excitatory neurotransmission by making glucose more available to neurons (Harley, 2007).

Neuroinflammation is a pathological feature of many neurodegenerative diseases, including AD (Perry et al., 2007; Heneka et al., 2015). Microglia are involved in neuroinflammation, with microglial activation occurring due to insults such as bacterial infection or circulating cytokines inducing a pro-inflammatory phenotype. The resultant cycle of cytokine release and activation of additional microglia leads to chronic neuroinflammation, increasing the risk of neurodegeneration. NE acting on microglia causes a suppression of proinflammatory cytokine signalling including IL-1, IL-6, TNF-α and inflammatory nitric oxide, through suppression of gene transcription (Mori et al., 2002), NE also upregulates gene transcription of anti-inflammatory molecules such as HSP-70 and MCP-1 in astrocytes and microglia (Heneka et al., 2010; Chalermpalanupap et al., 2013). The loss of LC neurons and the consequent reduction of NE anti-inflammatory signalling on neuroglia may thus be a likely contributor to the inflammation observed in brains with advanced Alzheimer’s disease [reviewed in (Arranz and de Strooper, 2019)].

Research also indicates an anti-inflammatory role for vagus nerve efferent signalling effects in the periphery, through acetylcholine release acting on tissue macrophages. This reduces cytokine synthesis and release similar to the effects on microglia. However, in the cholinergic anti-inflammatory pathway there is spleen involvement (Tracey, 2002), and further research will be required to establish whether the spleen is involved in the development of AD through Aβ accumulation (Yu et al., 2022) and whether VNS could improve the physiological capacity of the spleen to clear circulating Aβ. A reduction in cytokines including IL-6 and TNFα as a result of VNS has been measured in small-scale studies with patients with rheumatoid arthritis (Koopman et al., 2016), Crohn’s disease (Sinniger et al., 2020) and irritable bowel syndrome (Breit et al., 2018; Johnson and Wilson, 2018).

The peripheral effects of VNS are also highlighted by a study showing an interaction between the central sympathetic system and the parasympathetic VN to control arthritic joint inflammation (Bassi et al., 2017). iVNS in rats modulated arthritic joint inflammation through an afferent pathway mediated by LC activity. Afferent vagal stimulation activated two sympatho-excitatory brain areas, the paraventricular hypothalamic nucleus and the LC, the latter being essential for vagal control of arthritic joint inflammation. The authors showed that the LC could provide peripheral neuromodulation and reduced arthritic joint inflammation by increasing NE levels in the synovial fluid, leading to a reduction synovial inflammatory cytokines concomitant with a reduction of leukocytes in the synovial microcirculation. A reduction in peripheral inflammation may have an overall positive effect on the progression of AD (Perry et al., 2007).

Furthermore, it has been recently reported that in freely moving rats 2 h of iVNS -either a rapid stimulation cycle (7s on/18s off) or a standard stimulation cycle (30 s on/300 s off)- caused a significant reduction of body temperature (3^°^C and 1^°^C, respectively). This effect was sustained in animals treated with the NE neurotoxin DSP-4; thus, although the LC does not seem to mediate this effect, VNS could interact with other neurotransmitter systems, including cholinergic, GABAergic and serotonergic, and indirectly activate the hypothalamus which potentially could mediate the body cooling (Larsen et al., 2017). As these results show, it will be essential to consider the systemic impact on VNS to address their positive or negative contribution to memory modulation, AD pathology and any secondary effects.

## Neurons, Memory and Plasticity

Norepinephrine (NE) signals through G-protein coupled adrenergic receptors and its downstream effects can modulate the function of neuronal populations *via* effects on glia described above, or directly by synaptic mechanisms or changes in neuronal excitability. Neuronal effects of NE are highly varied between brain regions and adrenoreceptor subtype. For example, NE actions on α2-adrenoreceptors can increase network activity in the prefrontal cortex, but reduce excitatory transmission in neocortical and hippocampal pyramidal neurons, by limiting neurotransmitter release (O’Donnell et al., 2012).

Neuronal plasticity is a correlate of learning and memory. A widely researched mechanism of plasticity in hippocampal and cortical synapses involves the enhancement of synaptic efficacy through insertion of postsynaptic glutamate receptors of the AMPA subtype. Late-phase LTP in CA3-CA1 hippocampal synapses is a longer lasting potentiation dependent on protein synthesis. NE activates PKA which in turn phosphorylates AMPA receptors leading to their insertion in the plasma membrane (Hu et al., 2007). In the amygdala, LC-derived NE is hypothesised to consolidate emotionally stressful experiences by inducing late phase LTP (Hassert et al., 2004; Zuo et al., 2007). There is evidence that VNS can promote plasticity between the ventromedial prefrontal cortex and the basolateral amygdala, reflected by extinction of a conditioned fear response (Peña et al., 2014; Alvarez-Dieppa et al., 2016). Interestingly, selective optogenetic activation of LC-TH+ (dopamine releasing) neurons in mice enhanced synaptic function in the hippocampus and caused over 24 h persistence of an “everyday” type previously encoded memory, suggesting that LC stimulation can act as neuromodulator to promote the consolidation of hippocampal dependent memory. Thus, VNS may also allow native neuromodulation of memory pathways *via* dopaminergic signalling (Takeuchi et al., 2016).

The direct effects of NE acting on neurons are complex but appear to balance neuronal excitation and inhibition alongside neuroglial regulation [reviewed in (O’Donnell et al., 2012)]. Whether increased NE signalling induces potentiation or synaptic depression may depend on environmental factors, NE receptor subtype and intracellular signalling cascades specific to regions of the brain. Together, these effects modulate neuroplasticity in a region-specific manner tuned to autonomic regulation.

## Modulating Neuronal Circuits and Neuroinflammation With Vagus Nerve Stimulation

The positive effects of NE on neuroglial and neuronal cells suggest there is significant potential to modulate neuronal function, and neuroinflammation with VNS. In addition, VNS may also have positive impact on AD pathology. In AD mouse models that overexpress mutant human amyloid precursor protein (APP), LC lesions enhance Aβ pathology, inflammation and neuronal damage (Heneka et al., 2006; Kalinin et al., 2007). This suggests that NE signalling could be beneficial to slow down AD mechanisms. This is consistent with the observations of reduced glial activation and Aβ plaque pathology, and improved memory following increased availability of the NE precursor L-DOPS in the 5xFAD mouse model of AD (Kalinin et al., 2012).

In humans, iVNS has been used primarily to treat medication-resistant epilepsy. It significantly reduces interictal epileptiform discharges (Kuba et al., 2002) and can reduce seizure frequency and severity. In epilepsy patients with VNS implants, iVNS acutely improved memory performance and enhanced visual attention (Sun et al., 2017). In another study of 10 epilepsy patients using iVNS there was no overall effect on learning but an enhancement in consolidation, which improved memory retention (Ghacibeh et al., 2006). The authors suggest a mechanism of LC-induced activation of the amygdala, and enhanced LTP in the hippocampus. Working memory performance was hypothesised to underlie improved cognitive function overall and represents acute neuromodulation for the duration of active VNS. Patients with VNS implants for treatment-resistant depression have also showed rapid improvements in learning and memory within one month of stimulation, and these cognitive effects were sustained for up to two years of iVNS treatment (Desbeaumes Jodoin et al., 2018). Depression is frequently observed in patients with mild cognitive impairment (MCI) and is associated with faster progression to AD (Lyketsos et al., 2011). It is possible that VNS may be able to improve both cognition and depression symptoms, providing multiple benefits, but documenting these benefits will require reporting of neuropsychiatric symptoms in clinical trial outcomes.

Advances in the use of non-invasive tVNS for memory enhancement are also being made, as an alternative to invasive techniques. In one study with 60 participants, offline tVNS stimulation enhanced performance in working memory tasks (Sun et al., 2021). However, not all forms of short term memory may be enhanced by tVNS; in one study of 11 epilepsy patients with VNS implants, a decrease in figural memory was observed after high-intensity acute VNS (Helmstaedter et al., 2001) although this effect was acute and fully reversible. Due to a small sample size and lack of a double-blind placebo control, these results cannot be generalised but suggest the potential for effective modulation of memory via VNS in humans.

More recently, research is taking place using tVNS in healthy volunteers to study memory. Giraudier et al. (2020) report that high-confidence recognition memory was improved by a single-session of tVNS compared to sham stimulation, but overall word recognition and emotional word processing were not affected. A separate study reported improved response selection during sequential action in a group of 40 healthy participants (Jongkees et al., 2018). In another cohort, tVNS induced a higher accuracy on the verbal order memory task when applied to the ear tragus than either sham application to the ear lobe or in the absence of stimulation. The authors concluded that tVNS affects attention and cognition and that it is a potential method for modulating language and memory (Kaan et al., 2021).

While these results with tVNS are encouraging, modulation of memory with tVNS is not always replicable. An experiment by Mertens et al. (2020) found no effect of tVNS on verbal memory performance in either older or younger groups of healthy volunteers, failing to replicate the memory-enhancing effects seen in other research. One important factor that may contribute to contrasting effects of VNS on memory is differences in stimulation protocols, and the effects these may have on firing patterns in the LC. For example, most studies reporting success in memory outcome measures (Ghacibeh et al., 2006; Jongkees et al., 2018; Giraudier et al., 2020) report a 30 s on/off time of tVNS stimulation, whilst Mertens et al. (2020) only applied stimulation for 30 s during consolidation, with longer breaks in between. This indicates a regular “off” interval length may be important in regulating successful memory modulation. Broncel et al. (2020) highlight that the stimulation intensity for treatment of depression and epilepsy are different, therefore it is of outmost importance to optimise stimulation protocols in order to elicit effects on cognition and memory, especially with tVNS (Broncel et al., 2020). In the tVNS trials involving healthy volunteers, a stimulation frequency of 25 Hz and pulse width of 200–300 ms is commonly used.

A small number of studies examining VNS in AD have been completed, and several clinical trials are underway (Table 1). One early open label study (Sjögren et al., 2002) of 10 AD patients using iVNS showed that after 6 months of treatment 7 patients (70% of cohort) showed improvement or stability in cognitive measures [Alzheimer’s Disease Assessment Schedule (ADAS)-cog and the mini-mental state examination (MMSE)]. This study was extended with an additional 7 subjects and one year follow up (Merrill et al., 2006) and showed that after one year, 7 (41%) patients showed improvement or stability in the ADAS-cog, and 12 (71%) in both the MMSE and clinician impression of change (CIBIC +) scores. It is difficult to fully evaluate studies with no placebo arm, however, due to potential sensory reactions and the nature of iVNS, blind placebo designs may not be suitable here (Sun et al., 2017). These findings have encouraged larger scale studies and two exploratory randomised sham studies assessing the short term cognitive and physiological effects of non-invasive VNS are currently underway in a large group of mild cognitive impairment and AD subjects (see Table 1).

**TABLE 1:** Current clinical trials of VNS in Alzhemer’s disease and memory.

Trial name/Number	Patient population	Stimulation type	Outcome measures	Start/End
The wandering nerve: gateway to boost Alzheimer’s disease: https://ClinicalTrials.gov/show/NCT04908358	Older, healthy individuals *N* = 35	Active tVNS respiratory-gated 4 week Sham arm	Face-name association task up to 25 weeks after treatment Inflammatory blood biomarkers	August 2021/April 2026
The locus coeruleus and memory https://ClinicalTrials.gov/show/NCT02363504	Healthy older individuals and prodromal AD 60–85 years *N* = 35	Tesla magnetic resonance imaging (MRI) with memory task and tVNS	BOLD response during memory task Memory task performance (acute) NE levels	February 2017/December 2020 (overdue)
Modulating the locus coeruleus function https://ClinicalTrials.gov/show/NCT04877782	Healthy individuals 60–80 years *N* = 30	Auricular tVNS	Pupillometry BOLD response Memory task performance up to 10 days after tVNS	October 2021/January 2024
Treatment of mild cognitive impairment with transcutaneous vagal nerve stimulation (TVNS MCI) https://clinicaltrials.gov/ct2/show/NCT03359902	MCI patients, healthy older controls *N* = 125	Auricular tVNS	Delayed recall assessment	January 2018/May 2022

*N numbers represent per group.*

## Targeting Alzheimer’s Disease With Vagus Nerve Stimulation

Alzheimer’s disease (AD) is a progressive neurogenerative condition with a long prodromal phase. Patients are often diagnosed late in the disease progress, when the LC has already been subjected to pathological tau accumulation and degeneration. A recent study showed 55% LC neuron loss in postmortem samples from AD patients, however, only 30% LC neuron loss was observed in amnestic mild cognitive impairment patients (Kelly et al., 2017). Furthermore, a non-invasive study using neuromelanin-sensitive MRI technique to assess LC integrity in live patients showed that although there is a statistically significant reduction in signal intensity, 78% signal is still present in patients with mild to moderate AD (Hou et al., 2021). This suggests if vagal nerve stimulation intervention is used early in the disease process, LC activity dependent signalling mechanisms may still be available to vagus stimulation.

Vagal nerve stimulation has the potential to target multiple AD mechanisms and alter disease progression; it can support neuronal plasticity, alter the accumulation of Aβ pathology, enhance metabolic support of neuronal function *via* astrocytes and provide anti-inflammatory signalling. Furthermore, iVNS has a proven record of inhibiting epileptiform discharges (Kuba et al., 2002), and it has been observed that MCI or AD patients with epileptiform activity show an earlier age of onset of cognitive decline. This suggests that iVNS has the potential to provide further neuroprotection by preventing epilepsy in such patients and thus improve their clinical course (Vossel et al., 2013). Whether the beneficial antiepileptic effects of iVNS can be replicated with tVNS is unknown.

To answer whether VNS can function as a disease-modifying strategy using cognitive outcomes for AD will require a long-term study of 6-18 months, analysing cognitive scores with additional CSF or imaging-based biomarkers. Invasive devices are typically more clinically effective, require less user interaction once implanted and have greater potential for closed-loop interventions. Non-invasive devices have been recently selected as more suitable for clinical trials in AD (see Table 1) as they avoid the costs and complications associated with surgical implantation. This is particularly important when considering aged or cognitively impaired individuals, and taking into account prevalent co-morbidities in the target population. One such risk -which is higher in older individuals- is stroke, as implantation of an iVNS device requires manipulation of the carotid artery to expose the nerve, which can lead to migration of atheromatous plaques (van Lammeren et al., 2011). Comorbidies that may increase the risk of complications in this population include cerebrovascular diseases, synucleinopathies, tauopathies, frontotemporal lobar degeneration, and TDP-43–related diseases, reported in two-thirds of AD patients (Oosterveld et al., 2014; Katsumata et al., 2018).

The understanding of precise LC activity patterns evoked by specific iVNS or tVNS paradigms is very limited. Hulsey et al. (2017) used anesthesised rats to measure neuronal LC responses in response to direct VNS. They evaluated the LC responses to compare responses against a common parameter set used in animal research (16 pulses at 0.8 mA current amplitude, pulse width 100 μs, delivered at 30 Hz). They observed that even brief 0.5 s trains of 16 pulses at the much smaller current amplitude of 0.1 mA could elicit rapid, phasic neural activity in locus coeruleus neurons.

Epilepsy and depression iVNS uses a range of stimulation of 20–30 Hz, a pulse width of up to 500 μs, and stimulation on-time of 30–90 s followed by off-time of 5 min, stimulus intensity is in the range of 0.25–0.75 mA (Groves and Brown, 2005; Johnson and Wilson, 2018), however, the optimal parameters for modulation of cognition may differ from those routinely used for epilepsy or depression. Yap et al. (2020) have carried out a comprehensive review of stimulation parameters used in more than 60 tVNS studies. Although the submillisecond pulse width and 20–30 Hz stimulation range is shared with epilepsy stimulation, much higher current stimulations are required transcutaneously (typically up to 8 mA). An extremely broad set of stimulus regime parameters have been used, for example in two studies assessing tVNS in migraine one study used 90 s stimulation in total, while another study used 20 min twice daily for 12 weeks.

How the coupling of specific VNS stimulation paradigms to LC firing is translated into phasic or volume NE transmission, and the regimes necessary to elicit the protective NE effects on Alzheimer’s disease mechanisms in humans is unknown. Due to the variation in memory and physiological effects seen in iVNS, it is clear that frequency, intensity, stimulation interval and other aspects of stimulation protocols must be optimised to attain reproducible improvements in memory. After optimising these fundamental parameters, it might be possible to adjust VNS based on physiological feedback parameters such as electroneurogram activity (Sevcencu et al., 2016) in order to create a personalised treatment regime. This physiological feedback can also be applied to non-invasive VNS, with (Paleczny et al., 2021) and (Jacobs et al., 2020) recently reporting that stimulation improved memory scores with a larger effect size in respiratory-gated tVNS trials.

The results of ongoing trials will provide essential information on whether tVNS can provide cognitive improvement in AD patients. However, if these trials are unsuccessful or effect sizes too small to lead to significant improvement, it may be necessary to re-assess the stimulation paradigms. Furthermore, it may also be helpful to re-assess the potential of iVNS, and weigh the risks of this invasive procedure against the enhanced efficacy that may be achieved with direct stimulation of the nerve. The experimental and clinical evidence available suggest that VNS can target multiple mechanisms of Alzheimer’s disease with potential to modify the disease trajectory.

## Author Contributions

MV-C and BP conceived and designed the study. MV-C wrote the first draft of the manuscript. HW created figure. MV-C, RW, HW, BP, CH, and GC wrote sections of the manuscript. All authors contributed to the article and approved the submitted version.

## Conflict of Interest

BP: CEO and Founder—ElectronRx. The remaining authors declare that the research was conducted in the absence of any commercial or financial relationships that could be construed as a potential conflict of interest.

## Publisher’s Note

All claims expressed in this article are solely those of the authors and do not necessarily represent those of their affiliated organizations, or those of the publisher, the editors and the reviewers. Any product that may be evaluated in this article, or claim that may be made by its manufacturer, is not guaranteed or endorsed by the publisher.
